# MyelinJ: an ImageJ macro for high throughput analysis of myelinating cultures

**DOI:** 10.1093/bioinformatics/btz403

**Published:** 2019-05-16

**Authors:** Michael J Whitehead, George A McCanney, Hugh J Willison, Susan C Barnett

**Affiliations:** Institute of Infection, Immunity and Inflammation, College of Medical, Veterinary and Life Sciences, University of Glasgow, Glasgow, UK

## Abstract

**Summary:**

MyelinJ is a free user friendly ImageJ macro for high throughput analysis of fluorescent micrographs such as 2D-myelinating cultures and statistical analysis using R. MyelinJ can analyse single images or complex experiments with multiple conditions, where the ggpubr package in R is automatically used for statistical analysis and the production of publication quality graphs. The main outputs are percentage (%) neurite density and % myelination. % neurite density is calculated using the normalize local contrast algorithm, followed by thresholding, to adjust for differences in intensity. For % myelination the myelin sheaths are selected using the Frangi vesselness algorithm, in conjunction with a grey scale morphology filter and the removal of cell bodies using a high intensity mask. MyelinJ uses a simple graphical user interface and user name system for reproducibility and sharing that will be useful to the wider scientific community that study 2D-myelination *in vitro*.

**Availability and implementation:**

MyelinJ is freely available at https://github.com/BarnettLab/MyelinJ. For statistical analysis the freely available R and the ggpubr package are also required. MyelinJ has a user guide (Supplementary Material) and has been tested on both Windows (Windows 10) and Mac (High Sierra) operating systems.

**Supplementary information:**

Supplementary data are available at *Bioinformatics* online.

## 1 Introduction

Myelin is an essential component of the central nervous system (CNS) and its degeneration is associated with spinal cord injury and several CNS diseases, most notably multiple sclerosis (Goldenberg, 2012). Myelinating cultures generated from dissociated embryonic rodent spinal cords have been developed (Kerman *et al.*, 2015; Pang *et al.*, 2012; Sorensen *et al.*, 2008; Thomson *et al.*, 2008) as a tool to study developmental myelination (Ioannidou *et al.*, 2012), characteristics of spinal cord injury (Boomkamp *et al.*, 2012) and demyelination (Lindner and Linington, 2016). Consequently, myelinating cultures can be used as a high throughput screen for potential therapeutics that promote (re)myelination (McCanney *et al.*, 2018, 2019). One of the main bottlenecks for these screens is the accurate high throughput quantification of myelin sheaths. Currently, only a CellProfiler pipeline is freely available for analysis of *in vitro* myelination (available at https://github.com/muecs/cp). The MyelinJ ImageJ plugin we have developed is a freely available ImageJ (Schindelin *et al.*, 2012) macro that allows for high throughput analysis of individual experiments or large studies. The macro produces the percentage (%) of myelination and the % of neurite density for each image and links to R (R Core Team, 2013) for automated statistical analysis and graph production. The user friendly graphical user interface (GUI) and username system support reproducibility and sharing. MyelinJ aims to be widely applicable to the neuroscience community, because the settings can be easily adjusted/optimized specifically for your experiment. This study has only tested MyelinJ using myelinating cultures, however it is likely to also be useful for analysing slice cultures (Hill *et al.*, 2014) and tissue sections.

## 2 Materials and methods

Myelinating cultures were made according to Sorensen *et al.* (2008), demyelinated as described in McCanney *et al.* (2019) and manual analysis of micrographs was performed according to Sorensen *et al.* (2008). MyelinJ analyses % myelination and % neurite density of 2D fluorescent micrographs.

### 2.1 Background subtraction

Background is first subtracted either using ImageJ’s ‘rolling ball’ background subtraction, or using the neurite image as a mask to remove any bleed through of neurite fluorescence, followed by subtraction of pixels below a user provided threshold (the user can also select no background subtraction).

### 2.2 Myelin sheath selection

The Frangi vesselness (Frangi *et al.*, 1998) plugin is used to select myelin sheaths. Non-myelin sheath pixels are removed using a combination of ‘grey scale attribute filtering’ from the *MorphoLibJ* library (Legland *et al.*, 2016) and removal of a high intensity mask (that corresponds to cell bodies); both of which are optional. % myelination is calculated as:

Total myelin pixels/Total neurite pixels * 100.

### 2.3 Neurite selection

The percentage of (%) neurite density is calculated using the ImageJ filter ‘normalize local contrast’ (NLC). Alternatively, all standard ImageJ thresholding methods are also available to the user.

### 2.4 Neurite density analysis

% neurite density is calculated as:

Total neurite pixels/Total pixels * 100.

### 2.5 Statistical analysis

MyelinJ links ImageJ to R via the command line and uses the ggpubr package (https://github.com/kassambara/ggpubr) for statistical analysis and the production of publication ready graphs. MyelinJ performs Welch’s T test followed by correction for multiple testing using the false discovery rate (FDR). The user can choose between comparing all experimental conditions to each other or comparing all experimental conditions to control only.

## 3 Results

**Fig. 1.**
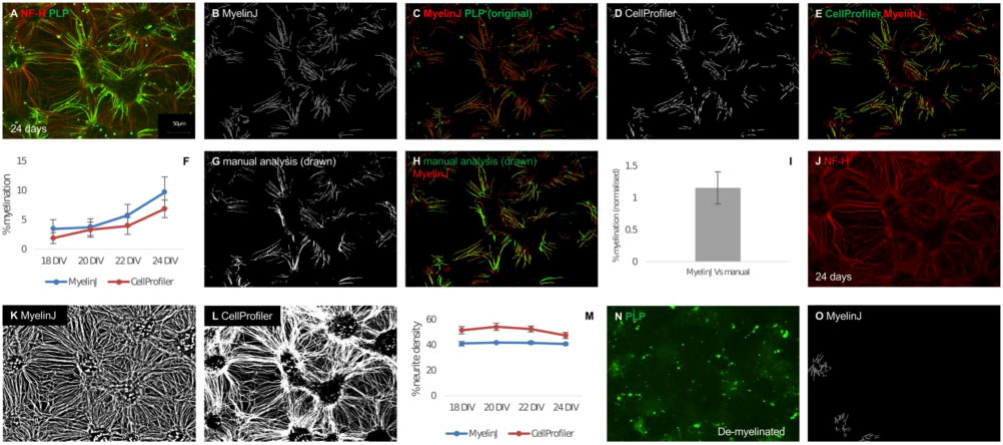
Application of MyelinJ using a myelinating time course and complement mediated demyelination. Representative images for myelination after 24 DIV are illustrated in this figure. Representative images of the full time course (18–24 DIV) are illustrated in Supplementary Figure S1. (**A**) Rat spinal cord myelinating culture after 24 DIV where SMI31-IR is red and PLP-IR is green. (**B**) MyelinJ myelin analysis of A. (**C**) Overlay comparing the original myelin (PLP-IR, in green) and the MyelinJ analysis (red). (**D**) CellProfiler analysis of A. (**E**) Overlay comparing the CellProfiler myelin analysis (green) to the MyelinJ myelin analysis (red). (**F**) Comparison between MyelinJ and CellProfiler for a % myelination time course of *in vitro* rat myelinating cultures between 18 and 24 DIV, calculated using PLP immunoreactivity as a percentage of NF-H immunoreactivity. (**G**) Manual analysis of A. (**H**) Comparison between manual analysis (green) and MyelinJ (red), for three images from 24 DIV. (**I**) Normalized % myelination difference between MyelinJ and manual analysis. (**J**) Representative image of Neurofilament heavy (NF-H, red) for A. (**K**) MyelinJ analysis of NF-H. (**L**) CellProfiler analysis of NF-H. (**M**) Comparison between MyelinJ and CellProfiler for a % neurite density time course of *in vitro* rat myelinating cultures between 18 and 24 DIV, calculated using NF-H immunoreactivity as a percentage of total pixels. (**N**) Representative image of an *in vitro* rat spinal cord myelinating culture after complement demyelination. (**O**) Print version all images are in black and white. MyelinJ analysis of N. Biological replicates = 1; technical replicates = 3 (Color version of this figure is available at *Bioinformatics* online.)

MyelinJ calculates ‘% myelination’ and ‘% neurite density’. In order to test the macro we first analysed an *in vitro* myelinating time course and compared it to the freely available CellProfiler pipeline (Fig. 1A–E, Supplementary Fig. S1 illustrates comparisons for the entire time course). MyelinJ is able to identify about 46% more myelin sheath pixels on average per image than Cellprofiler, based on a comparison of 30 images at each time point taken from three technical replicates. In previously published data based on this model this is the standard number of images to constitute a biological replicate (Fig. 1E and F and Supplementary Fig. S1C). MyelinJ’s neurite density is also more consistent across images (Fig. 1J–M and Supplementary Fig. S4). MyelinJ was also compared to manual analysis of % myelination by drawing over the myelin sheaths (Fig. 1G–I). MyelinJ is very similar to manual analysis and takes ∼15–18 s per image compared to about 15 min per image. Furthermore, we find that Frangi vesselness is superior to the Otsu thresholding method in ImageJ for determining % myelination, as illustrated in Supplementary Figure S1E.

MyelinJ’s ability to ignore non-myelin sheath background was tested using myelinating cultures that have been demyelinated in a complement-mediated manner (McCanney *et al*, 2019). Demyelination leaves significant background, which is predominantly not myelin sheaths (Fig. 1N). MyelinJ effectively disregards the non-myelin sheath background (Fig. 1M). Furthermore, MyelinJ successfully analyses the remaining undamaged myelin sheaths (Fig. 1M). In comparison, CellProfiler misses the majority of these myelin sheaths (for Fig. 1N CellProfiler identified 0% myelination—image post analysis not shown). Supplementary Figure S2 illustrates the graphs and statistical analysis performed by MyelinJ for an example dataset (demyelination versus remyelination), using the ggpubr package in R.

## 4 Conclusions

This newly developed MyelinJ is a user friendly ImageJ macro for the analysis of fluorescent micrographs of 2D myelinating cultures providing quantification of the % of neurite density and the % of myelination. To the best of our knowledge, there are currently no other publicly available ImageJ macros for this analysis and MyelinJ marks a significant improvement upon the freely available CellProfiler pipeline (available at https://github.com/muecs/cp), being able to more accurately analyse both myelin sheaths and neurites. MyelinJ also offers automated calculation of % neurite density and % myelination in order to avoid human error, can analyse complex experiments where a summary of the results for each condition is provided and seamlessly links to R for the graphical representation of results and statistical analysis. In addition, MyelinJ analyses myelin sheaths significantly better than the Otsu ImageJ thresholding algorithm (Otsu was selected following comparisons between all ImageJ thresholding algorithms). This is the first time (to the best of our knowledge) that an ImageJ macro that can interact with the statistical package R has been made freely available. MyelinJ uses the ggpubr package to perform statistical analysis and produce publication quality graphs, providing a seamless analysis pipeline from raw images to graphical representation and statistical analysis for high throughput screens.

## Author contributions

M.J.W. conceived the study, wrote the main manuscript text and prepared all figures. G.M. performed all of the rat myelinating spinal cord culture experiments. S.C.B. and H.J.W. directed the study and wrote the manuscript. All authors reviewed, edited and approved the final manuscript.

## Funding

This work was supported by a Doctoral Training Grant to M.J.W. provided jointly by the UK MRC [MR/K501335/1] and UK BBSRC [BB/J013854/1], the Wellcome Trust [202789/Z/16/Z], Medical Research Scotland (MRS; G.M.) and MS society of Great Britain (56).

*Conflict of Interest*: none declared.

## Supplementary Material

btz403_Supplementary_Data
