# Supplementary material for: MyelinJ: an ImageJ macro for high throughput analysis of myelinating cultures
Source: Bioinformatics. 2019 May 16;35(21):4528–30. doi: 10.1093/bioinformatics/btz403 (PMC6821319; doi:10.1093/bioinformatics/btz403)
Supplement: btz403_Supplementary_Data [file btz403_supplementary_data.zip › btz403-suppl_data/MyelinJ usermanual revised.pdf]

MyelinJ User Manual

April 1, 2019

Michael J. Whitehead, George McCanney, Hugh J. Willison, Susan C. Barnett*

Institute of Infection, Immunity and Inflammation, College of Medical, Veterinary and Life Sciences, University of Glasgow, 120 University Place, Glasgow G12 8TA, UK

*To whom correspondence should be addressed

Professor Susan C Barnett
University of Glasgow, Institute of Infection, Immunity and Inflammation, College of Medical, Veterinary and Life Sciences, GBRC, Room B329,
120 University Place, Glasgow, G12 8TA.

Telephone: 44 (0)141 330 8409 E-mail: Susan.Barnett@Glasgow.ac.uk

Contents

1 Overview **3**

2 Installation **4**

2.1 Requirements 4

2.2 Download Fiji and MorphoLibJ 4

2.3 Download MyelinJ 5

2.4 Download R for statistical analysis 8

3 Running MyelinJ **10**

4 Statistical analysis **16**

1 Overview

MyelinJ is an ImageJ macro for the high throughput analysis of 2D fluorescent micrographs of myelination. MyelinJ has been designed and tested for the analysis of *in vitro* myelinating spinal cord cultures, however it may also be useful for other applications. The output of this analysis is % neurite density and % myelination. MyelinJ has a user friendly graphical user interface (GUI) that allows the user to change and visualise settings for optimal analysis. In addition, there is a user name system so that different settings can be stored and shared easily. MyelinJ can analyse everything from one micrograph to a complex experiment with several experimental conditions. For complex experiments each condition is analysed separately and a summary sheet with the average values for each condition is displayed. MyelinJ also links to R for statistical analysis and the production of graphs illustrating value distribution for each image as well as publication quality graphs with averages and $*$ for p values displayed.

2 Installation

2.1 Requirements

- **Operating system:** MyelinJ works on both Windows and Mac operating systems (tested on Windows 10 and macOS High Sierra).
- **Image format:** Merged 2D neurite and myelin fluorescent microscope images in .tif format (other fluorescent channels can also be present, but will be ignored by the analysis). Images must have unique names (but can be in any number of subdirectories [folders within folders]).
- **Software:** The latest version of FIJI (last tested on version 2.0.0-rc-69/1.52i). For statistical analysis the latest version of R is required (last tested on version 3.5.1).
- **Folder structure** For analysing multiple experimental conditions, a directory (main folder) containing subdirectories (folder) for each experimental condition is required. Within the subdirectories for each experimental conditions the images can be in any number of folders. If multiple experimental conditions are not being analysed then all of the images should be placed in one folder. Within this folder there can be any number of subdirectories (folders within folders).

2.2 Download Fiji and MorphoLibJ

Download the latest version of Fiji at https://imagej.net/Fiji/Downloads. Open Fiji and download MorphoLibJ by going to "Help >Update.." **(1)**, click on "Manage update sites" **(2)** and then "IJPB-plugins" **(3)**. Press Close on "Manage update sites", press "Apply changes" **(2)** and then restart FIJI.

1

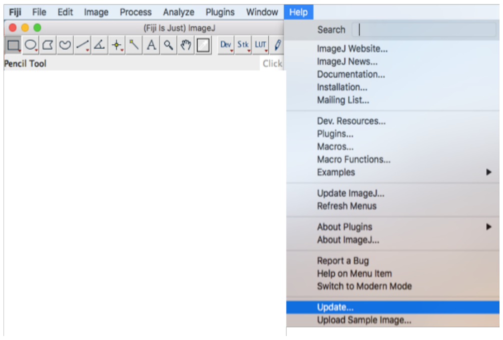

2

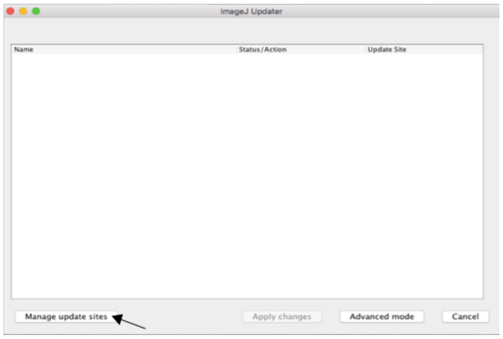

3

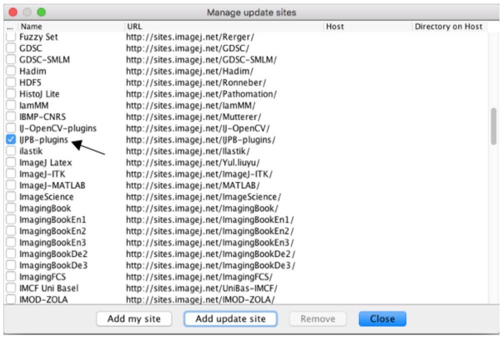

2.3 Download MyelinJ

MyelinJ is freely available on GitHub. In order to download go to the following website: `https://github.com/BarnettLab/MyelinJ`. On the "Clone or download' button press "Download zip" (**1**).

For macOS simply double click on the downloaded .zip file (found in the downloads folder. Filename: "MyelinJ-master.zip") and copy the new folder called "MyelinJ-master" (**2**). You then need to find the plugins file in Fiji. For macOS Fiji will be found in the "Applications" folder (**3**). Once you have found the Fiji icon press ctrl and left click the Fiji icon then click "Show Package Contents" (**3**). Put the unziped folder (which should be called MyelinJ-master) into the "plugins" folder. (**4,5**).

For Windows right click on the downloaded .zip (found in the downloads folder. Filename: "MyelinJ-master.zip") file and press "Extract All". Once extracted open the "MyelinJ-master.zip" folder and copy the "MyelinJ-master" folder (**2**). Find the Fiji folder on your computer (likely saved in the programs file - specified during installation). Open the Fiji folder and place the "MyelinJ-master" folder in the folder called "plugins"" (**4,5**).

1

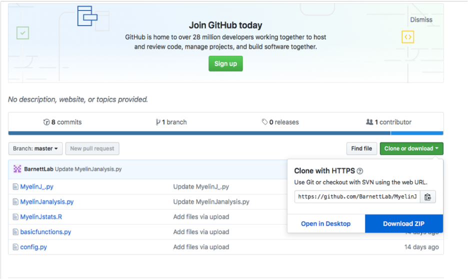
Join GitHub today
GitHub is home to over 28 million developers working together to host and review code, manage projects, and build software together.
Sign up
Dismiss
No description, website, or topics provided.
8 commits
1 branch
0 releases
1 contributor
Branch: master
New pull request
Find file
Clone or download
BarnettLab Update MyelinJanalysis.py
MyelinJ_.py
Update MyelinJ_.py
MyelinJanalysis.py
Update MyelinJanalysis.py
MyelinJstats.R
Add files via upload
basicfunctions.py
Add files via upload
config.py
Add files via upload
14 days ago
Clone with HTTPS
Use Git or checkout with SVN using the web URL.
Open in Desktop
Download ZIP

2

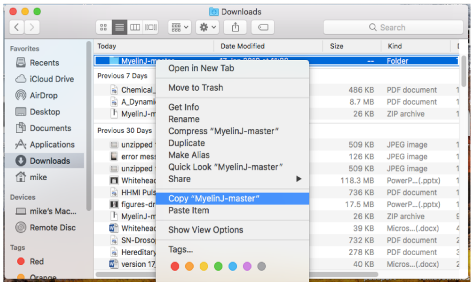
Downloads
Search
Favorites
Recents
iCloud Drive
AirDrop
Desktop
Documents
Applications
Downloads
mike
Devices
mike's Mac...
Remote Disc
Tags
Red
Orange
Today
Date Modified
Size
Kind
Folder
Previous 7 Days
Previous 30 Days
Open in New Tab
Move to Trash
Get Info
Rename
Compress "MyelinJ-master"
Duplicate
Make Alias
Quick Look "MyelinJ-master"
Share
Copy "MyelinJ-master"
Paste Item
Show View Options
Tags...
486 KB
PDF document
8.7 MB
PDF document
26 KB
ZIP archive
509 KB
JPEG image
126 KB
JPEG image
509 KB
JPEG image
118.3 MB
PowerP...(.pptx)
736 KB
PDF document
17.5 MB
PowerP...(.pptx)
26 KB
ZIP archive
39 KB
Micros...(.docx)
732 KB
PDF document
278 KB
PDF document
40 KB
Micros...(.docx)

3

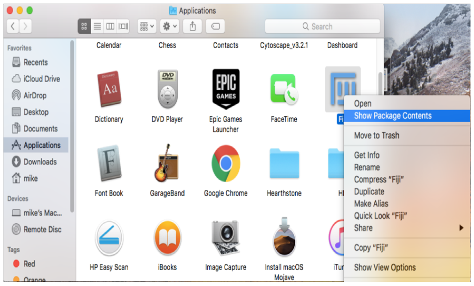
Applications
Search
Favorites
Recents
iCloud Drive
AirDrop
Desktop
Documents
Applications
Downloads
mike
Devices
mike's Mac...
Remote Disc
Tags
Red
Orange
Calendar
Chess
Contacts
Cytoscape_v3.2.1
Dashboard
Dictionary
DVD Player
Epic Games Launcher
FaceTime
Font Book
GarageBand
Google Chrome
Hearthstone
HP Easy Scan
iBooks
Image Capture
Install macOS Mojave
Open
Show Package Contents
Move to Trash
Get Info
Rename
Compress "Fiji"
Duplicate
Make Alias
Quick Look "Fiji"
Share
Copy "Fiji"
Show View Options

4

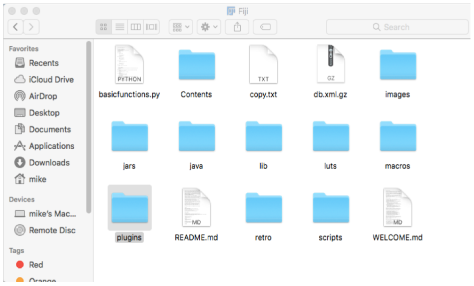
Fiji
Search
Favorites
Recents
iCloud Drive
AirDrop
Desktop
Documents
Applications
Downloads
mike
Devices
mike's Mac...
Remote Disc
Tags
Red
Orange
basicfunctions.py
Contents
copy.txt
db.xml.gz
images
jars
java
lib
luts
macros
plugins
README.md
retro
scripts
WELCOME.md

5

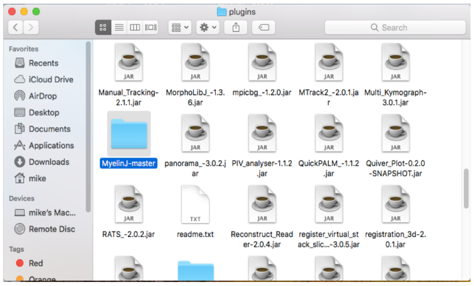
plugins
Search
Favorites
Recents
iCloud Drive
AirDrop
Desktop
Documents
Applications
Downloads
mike
Devices
mike's Mac...
Remote Disc
Tags
Red
Manual_Tracking-2.1.1.jar
MorphoLibJ_-1.3.6.jar
mpicbg_-1.2.0.jar
MTrack2_-2.0.1.jar
Multi_Kymograph-3.0.1.jar
MyelinJ-master
panorama_-3.0.2.jar
PIV_analyser-1.1.2.jar
QuickPALM_-1.1.2.jar
Quiver_Plot-0.2.0-SNAPSHOT.jar
RATS_-2.0.2.jar
readme.txt
Reconstruct_Reader-2.0.4.jar
register_virtual_stack_slic...-3.0.5.jar
registration_3d-2.0.1.jar

2.4 Download R for statistical analysis

If you want MyelinJ to perform statistical analysis you also need to download the free statistical package R. To download R go to https://cran.r-project.org/ , click “Download R” for your operating system. For Windows (**1**) click base, click download, right click the install file and follow installation instructions. For Mac (**2**) click on the blue .pkg file link to the left of latest releases and follow installation instructions.

1

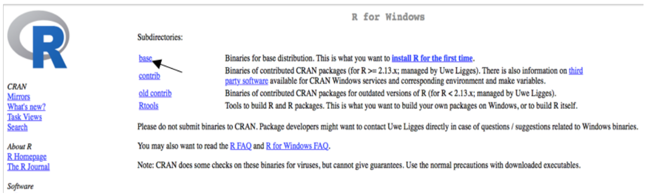

2

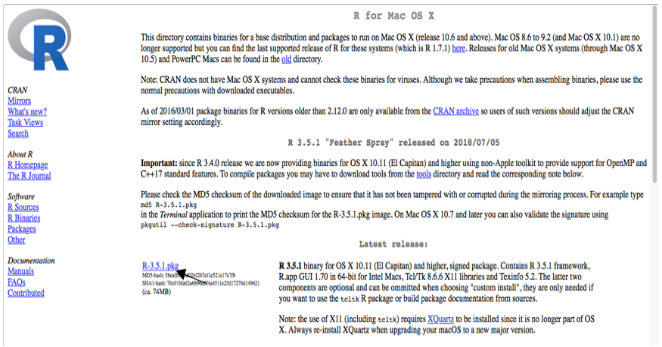

You will also need to download a package called ggpubr (https://github.com/kassambara/ggpubr). If you would like to use R in the future, the free integrated development environment (IDE) R Studio is recommended (https://www.rstudio.com/). Click on your newly download R (or R studio) and in the console type the below lines separately and press enter (**1**); you will see ggpubr being downloading. (note - without R studio you will be asked to choose a mirror for this session (**2**). You can select any mirror you want (independent of your physical location).

```
install.packages("devtools")

devtools::install_github("kassambara/ggpubr")
```

1

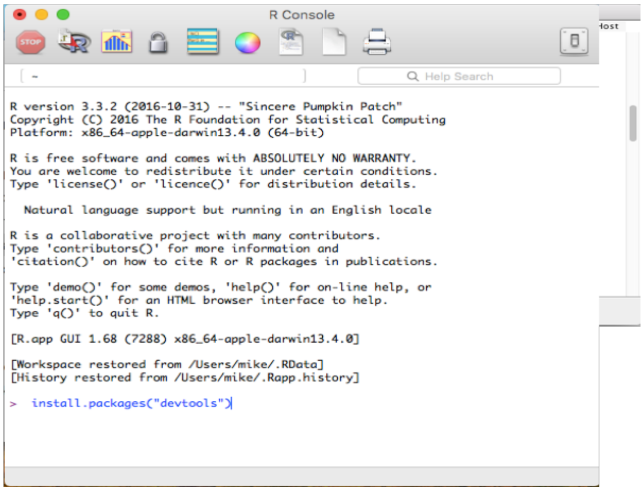
R Console
Help Search
R version 3.3.2 (2016-10-31) -- "Sincere Pumpkin Patch"
Copyright (C) 2016 The R Foundation for Statistical Computing
Platform: x86_64-apple-darwin13.4.0 (64-bit)
R is free software and comes with ABSOLUTELY NO WARRANTY.
You are welcome to redistribute it under certain conditions.
Type 'license()' or 'licence()' for distribution details.
Natural language support but running in an English locale
R is a collaborative project with many contributors.
Type 'contributors()' for more information and
'citation()' on how to cite R or R packages in publications.
Type 'demo()' for some demos, 'help()' for on-line help, or
'help.start()' for an HTML browser interface to help.
Type 'q()' to quit R.
[R.app GUI 1.68 (7288) x86_64-apple-darwin13.4.0]
[Workspace restored from /Users/mike/.RData]
[History restored from /Users/mike/.Rapp.history]
> install.packages("devtools")

2

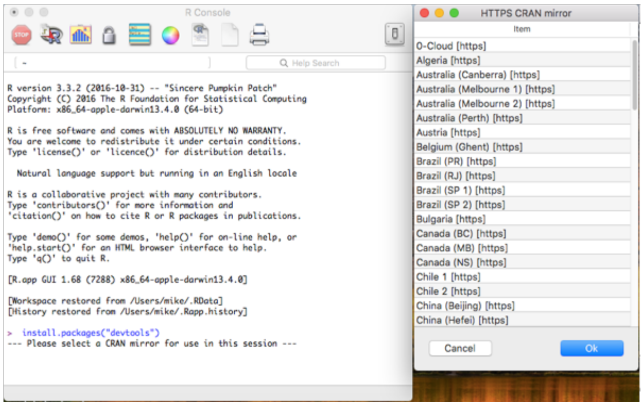
R Console
Help Search
R version 3.3.2 (2016-10-31) -- "Sincere Pumpkin Patch"
Copyright (C) 2016 The R Foundation for Statistical Computing
Platform: x86_64-apple-darwin13.4.0 (64-bit)
R is free software and comes with ABSOLUTELY NO WARRANTY.
You are welcome to redistribute it under certain conditions.
Type 'license()' or 'licence()' for distribution details.
Natural language support but running in an English locale
R is a collaborative project with many contributors.
Type 'contributors()' for more information and
'citation()' on how to cite R or R packages in publications.
Type 'demo()' for some demos, 'help()' for on-line help, or
'help.start()' for an HTML browser interface to help.
Type 'q()' to quit R.
[R.app GUI 1.68 (7288) x86_64-apple-darwin13.4.0]
[Workspace restored from /Users/mike/.RData]
[History restored from /Users/mike/.Rapp.history]
> install.packages("devtools")
--- Please select a CRAN mirror for use in this session ---
HTTPS CRAN mirror
Item
0-Cloud [https]
Algeria [https]
Australia (Canberra) [https]
Australia (Melbourne 1) [https]
Australia (Melbourne 2) [https]
Australia (Perth) [https]
Austria [https]
Belgium (Ghent) [https]
Brazil (PR) [https]
Brazil (RJ) [https]
Brazil (SP 1) [https]
Brazil (SP 2) [https]
Bulgaria [https]
Canada (BC) [https]
Canada (MB) [https]
Canada (NS) [https]
Chile 1 [https]
Chile 2 [https]
China (Beijing) [https]
China (Hefei) [https]
Cancel
Ok

3 Running MyelinJ

1. Open FIJI and select MyelinJ from the plugins tab (it will be near the bottom of the list and is called MyelinJ-master). **Note for Windows users** – an error message (console: "Failed to install ": java.nio.charset.Uns upportedCharsetException: cp0") comes up the first time MyelinJ is run in a new instance of ImageJ. The error message can be ignored – it is a Windows bug.

2. A file explorer will open so that the folder containing the images to be analysed can be selected. Refer to section 2.1 for required folder structure.

3. Click new user. Click "Multiple experimental conditions?" if required and click "Perform statistical analysis" if you would like MyelinJ to perform statistical analysis. If multiple experimental conditions are analysed without statistical analyses, each experimental condition will be analysed separately and a summary sheet with result averages will also be produced. **Note** - for statistical analysis you must have followed the instructions in section 2.4.

4. press OK (once made, user names can be selected from the drop down menu and pressing OK will run the analysis). **Note** - all user settings are saved except for the parameters: "Multiple experimental conditions?" and "Perform statistical analysis?" which must be specified prior to each analysis by clicking the checkbox prior to pressing OK.

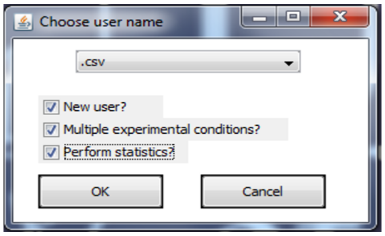

5. (**optional**) Define experimental conditions for statistical analysis.

   (a) Check the file path for the R program files is correct. The default file path for the OS being used (Windows or Mac (macOS High Sierra) will be displayed.The version of R may also need to be changed.

   (b) Enter the number of experimental conditions required and press Enter on the keyboard.

   (c) Enter the name of each experimental condition in the empty boxes above each column (these names will be displayed on the graphs). The left hand column denotes folder names for each experimental repeat and condition. Use the check boxes to select which experiment belongs to which experimental condition and press OK. **Note** - if you

want the statistical analysis to compare all experimental conditions to only the control (rather than comparing all experimental conditions to other) type "(control)" next to the name of the experimental condition without any spaces e.g. D1(control). "(control)" will be removed from the name on the graph.

(d) Press OK to continue defining analysis settings or to start the analysis if predefined settings were already selected.

(e) If you would like to change the number of experimental conditions first press Reset and then enter the new number of experimental conditions.

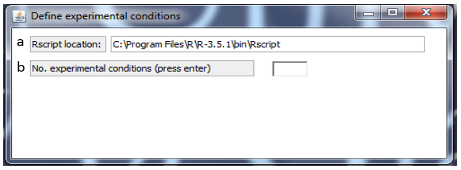

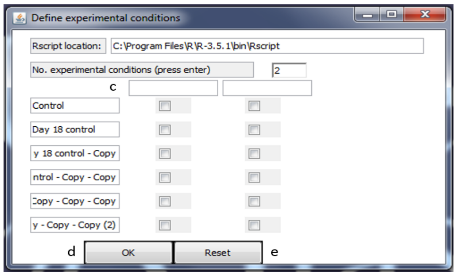

6. Enter a unique user name and press OK. **Note** – if the user name already exists the user will be asked whether to overwrite with new settings.

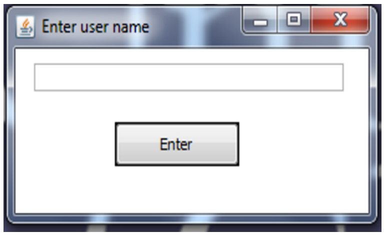

7. Define channels used for neurites and myelin in the images to be analysed and press enter (Figure 1C). **Note** – this analysis assumes that the images for myelin and neurites have been merged together (refer to section 2.1).

8. Define settings for the myelin channel. These settings include, background subtraction to remove noise, selecting a thresholding algorithm to convert the image to binary, a method for removing PLP-IR cell bodies and a grey

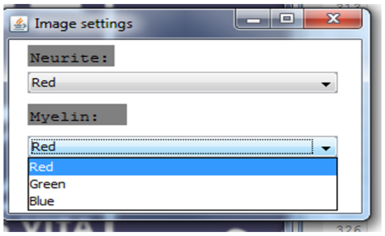

scale filter to remove background. The first image in the selected folder will be displayed. Click the next image button on the bottom right to open the next image in the folder for comparison (the next image will be displayed above the previous). Alternatively open an image of your choosing (using ImageJ) and then click the “Use selected image” checkbox in order to use the image. To revert back to using images from the selected folder(s) simply uncheck the box. Where values are to be entered, you can alter the value as many times as required to identify the optimal value.

Note - all of these settings are optional. If no options are selected only the Frangi vesselness algorithm will be used to analyse % myelination.

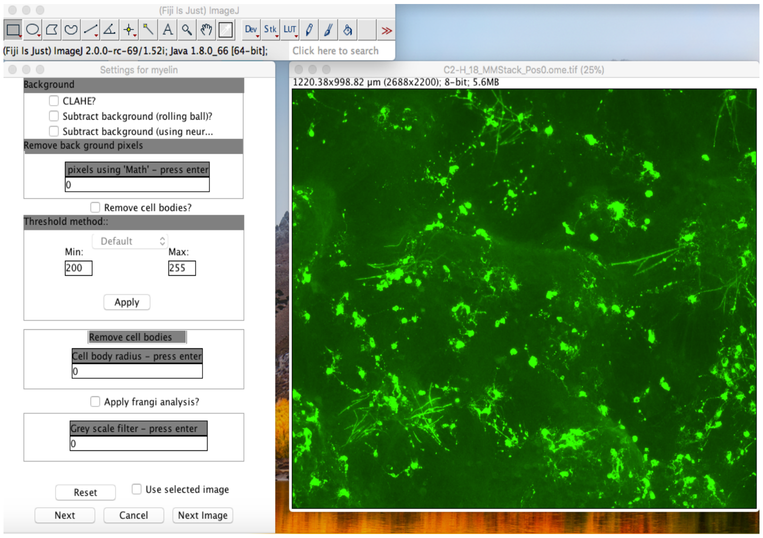

(a) **Enhancement/background subtraction**. Click CLAHE to use enhance local contrast (refer to https://imagej.net/Enhance_Local_Contrast_(CLAHE)). This can be used alone or in combination with either “rolling ball” or ”using neurites”. “rolling ball” uses ImageJ’s “Subtract Background” function (refer to http://imagejdocu.tudor.lu/doku.php?id=gui:process:subtract_background). “using neurites” subtracts the neurite channel (without alteration) from the myelin channel. This is useful for bleed through between channels.

"using neurites" in combination with "subtract pixels using Math" works very well for removing bleed through and background in our images (refer to http://imagejdocu.tudor.lu/doku.php?id=gui:process:math).

(b) **Remove cell bodies**. To remove cell bodies first click the checkbox "remove cell bodies?". **Thresholding**: Select optimal threshold method and how the histogram should be cropped ("ImageJ>Toolbar >Image>Adjust>Threshold" on ImageJ's menu bar should be used to identify optimal threshold method and parameters for cropping the histogram). Enter the optimal settings (checking multiple images to find the optimal average) into the dialogue box and press apply. Thresholding should select high intensity cell bodies only (We found the Default threshold method and cropping the histogram between 100-150 and 200 worked well for our images). Refer to https://imagej.net/Auto_Threshold for more information on thresholding options. Selection of the "best" threshold algorithm is relatively subjective. "threshold check" from the BioVoxxel toolbox (https://imagej.net/BioVoxxel_Toolbox#Threshold_Check, download instructions at the top of the page) is a useful guide for selection. In our experience we find thresholding cell bodies to be relatively easy as they are usual very bright, in comparison to the rest of the image.

Cell body radius uses ImageJ's remove outliers function (refer to http://imagejdocu.tudor.lu/doku.php?id=gui:process:noise. Enter value and press enter (10 was a good starting point for images tested). This filters the thresholded cell bodies to ensure no myelin sheaths are detected. If thresholding alone is optimal leave at 0.

(c) **Apply Frangi vesselness**. Frangi vesselness analysis will performed after background subtraction and removal of cell bodies (if these settings have been defined).

(d) **Minimum value - press enter**. This uses the grey scale morphology filter from the MorphoLibJ library (refer to https://imagej.net/MorphoLibJ). Briefly, the minumum value corresponds to the length of the dialogonal of the bounding box. This helps to differentiate between circular (cell bodies) and elonganted (myelin sheaths) structures. Values between about 40 and 80 worked well for our images.

(e) The reset button will close all open images and remove all settings. Next will open for the next dialogue box for neurite settings. Cancel will close everything and cancel the macro.

9. **Define settings for dense neurites.** These settings include, normalise local contrast (NLC) to convert the image to binary and Despeckle (refer to http://imagejdocu.tudor.lu/doku.php?id=gui:process:noise) to remove background noise. Press next image to view the settings on another image. Alternatively open an image of your choosing (using ImageJ) and

then click the "Use selected image" checkbox in order to use the image. To revert back to using images from the selected folder(s) simply uncheck the box. Where values are to be entered, you can alter the value as many times as required to identify the optimal value.

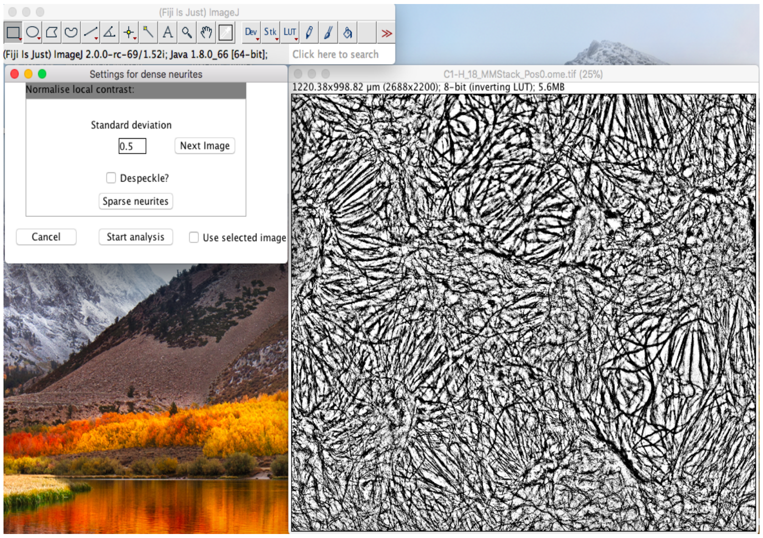

(a) **Normalise local contrast**. If the displayed image does not look optimal enter a new value and press enter on the keyboard for the image to be displayed. This value is for standard deviations and is used as an estimate for the deviation in pixel intensities throughout the image (the smaller the value the greater the contrast between background and object).

(b) **Despeckle**. Press despeckle to run ImageJ's despeckle function which is a median filter for the removal of "salt and pepper" noise.

(c) **Alternative settings**. If normalise local contrast is not optimal for thresholding of neurites, press the sparse neurites button for alternative settings (see below).

10. **Define settings for sparse neurites**. Click the sparse neurites button. These settings include, background subtraction to remove noise, image enhancement using CLAHE and several thresholding algorithms. Press next image to view the analysis settings on another image in the selected file. Where values are to be entered, you can alter the value as many times as required to identify the optimal value.

    (a) Background subtraction using ImageJ's "Background Subtraction" method, which uses the "rolling ball" algorithm.

    (b) Image can be enhanced using enhance local contrast (CLAHE) (or none).

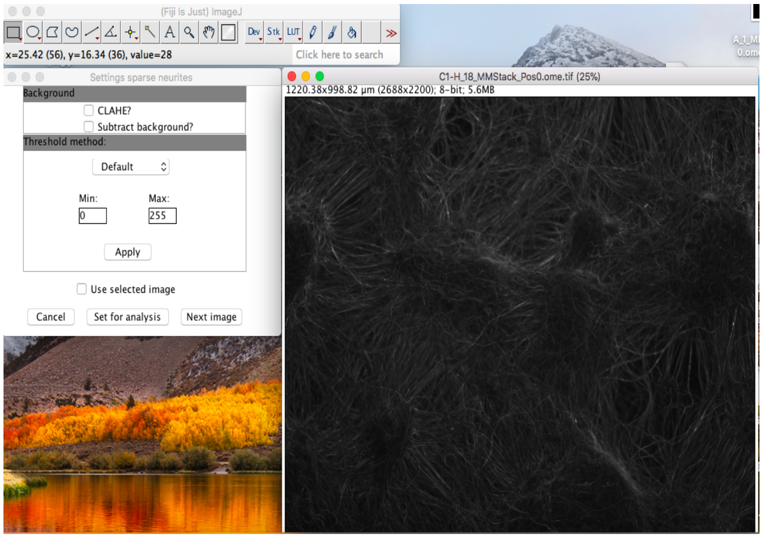

(c) The thresholding and histogram settings can then be selected – as for 8b.

(d) Pressing the set for analysis button means the settings entered will be used by the macro. The dialogue box will close and the previous dialogue box will open ("settings for dense neurites"). The "Start analysis" button can then be pressed to start the analysis. Alternatively, pressing cancel will close the sparse neurites dialog box and the macro will use settings for dense neurites.

11. **Run analysis**. Once you are happy with all of the settings press start analysis. Once the analysis has finished you will be prompted by a message.

12. **Results**. For each image analysed the thresholded myelin and neurites images are saved, so that the user can check MyelinJ is analysing the images approriately. For each folder of images a comma separated values (csv) file called "Result.csv" is produced. This file can be opened using any spreadsheet application. The file contains % myelination and % neurite density of each image analysed in the folder (denoted by the images name). % neurite density is calculated as: Total image pixels\total neurite pixels * 100 and % myelination is calculated as: Total neurite pixels\total myelin pixels * 100. If multiple experimental conditions have been analysed then a sheet called "Result-Summary.csv" will also be produced. This file contains the average % myelination and % neurite density for each experiment (defined as each subfolder within the main folder - refer to section 2.1). For statistics refer to section 4.

13. **Future analysis**. The next time MyelinJ is opened the user name can

be selected from a dropdown box on the "choose username" dialog box (section). Pressing OK will run the analysis (refer to numbers 3 and 4). (**Note** - remember to select multiple experimental conditions? and perform statistics? if required, as these settings are not saved).

4 Statistical analysis

MyelinJ can link to the freely available statistics package R (refer to section 2.4 for download instructions and refer to section 3.5 for instructions on defining experimental conditions for analysis). MyelinJ uses ggpubr (refer to `https://github.com/kassambara/ggpubr`), which has been specifically designed for ease of use. For statistical analysis MyelinJ uses a T test, that uses the false discovery rate (FDR) for multiple comparison corrections. Welch's T test is used by defualt, which means experimental conditions can have a varying number of repeats (in comparison to the Student's T test where each experimental condition has to have the same number of repeats). By default MyelinJ will perform a T test for every possible comparison (with multiple test correction if required). MyelinJ can also compare all experimental conditions to control only (refer to section 3.5c for instructions). This will produce a bar graph (for % myelination and % neurite density) where each experimental condition is compared, any significant differences ($p<0.05$) will be denoted by comparison lines and the appropriate number of * to represent p values. Each experimental repeat value average is plotted as a dot and the error bars denote standard deviation. The colours of the bar graph are defined by the nature publishing group (NPG) palette from the ggsci package (`https://cran.r-project.org/web/packages/ggsci/vignettes/ggsci.html`). In addition, bar graphs will also be produced where the value from each individual image is plotted, so that the variation within an experiment can also be easily visualised. MyelinJ will also save csv files which define all of the comparisons perform, the statistical analyses used and absolute p values (as produced by the ggpubr package).
