# Supplementary material for: MyelinJ: an ImageJ macro for high throughput analysis of myelinating cultures
Source: Bioinformatics. 2019 May 16;35(21):4528–30. doi: 10.1093/bioinformatics/btz403 (PMC6821319; doi:10.1093/bioinformatics/btz403)
Supplement: btz403_Supplementary_Data [file btz403_supplementary_data.zip › btz403-suppl_data/supplementary figures.pdf]

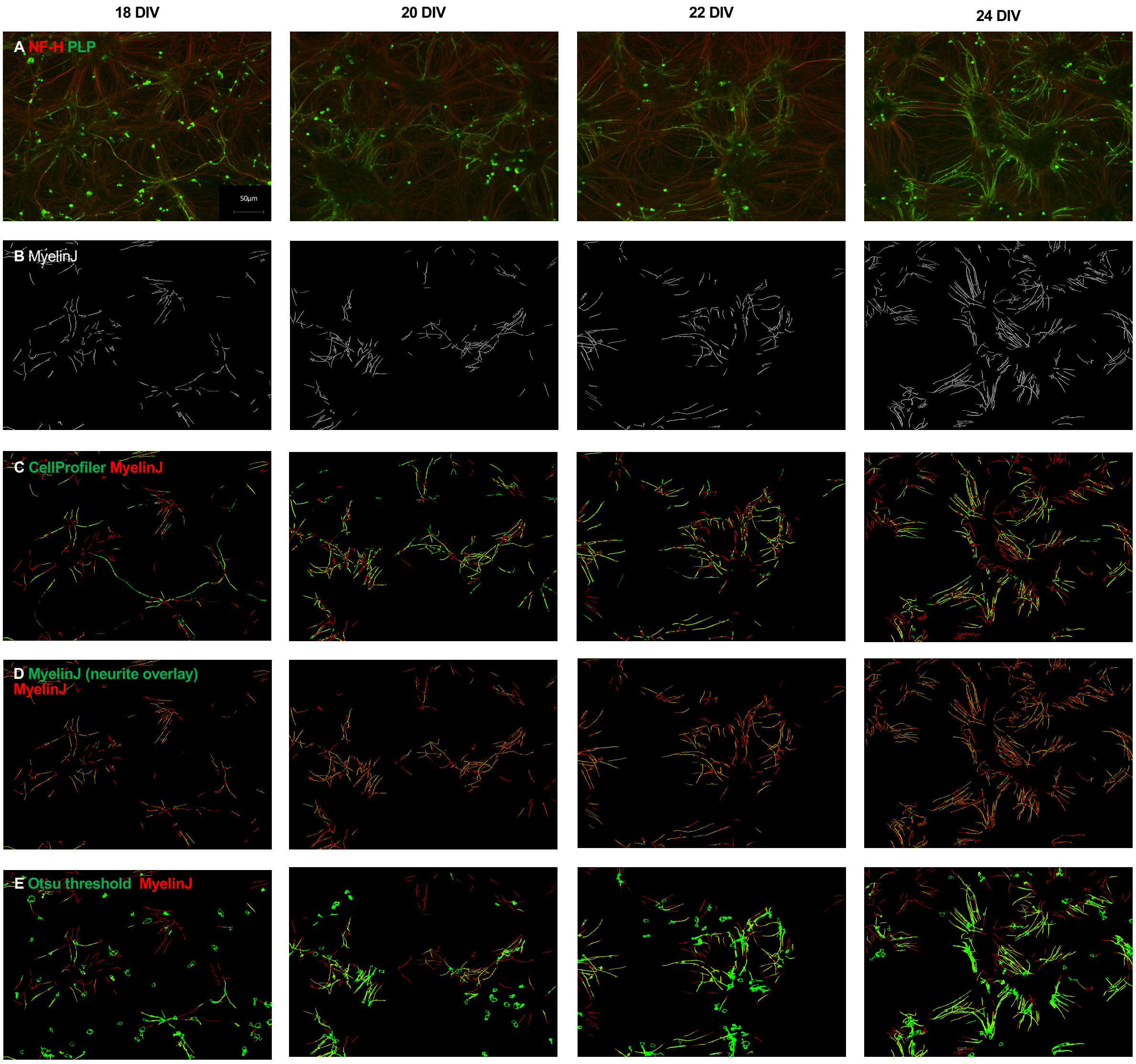

Supplementary figure 1: Comparing thresholding methods for myelin sheath quantification using an *in vitro* myelinating timecourse. **A)** Rat myelinating spinal cord cultures from 18-24 DIV where NF-H is red and PLP is green. **B)** MyelinJ analysis for A. **C)** CellProfiler analysis for A in green compared to MyelinJ analysis of A in red. **D)** MyelinJ analysis of A in green where overlapping neurite and myelin pixels only are shown, compared to normal MyelinJ analysis of A in red. Normal MyelinJ analysis identified a lot more myelin sheaths but also outlines some cell bodies not seen with the pixel overlap. **E)** Looking at all ImageJ's thresholding methods we identified "Otsu" as the most promising. Compared to MyelinJ "Otsu" identifies more cell bodies, less myelin sheaths and does not deal with changes in pixel intensity as well. Biological replicates = 1, technical replicates = 3.

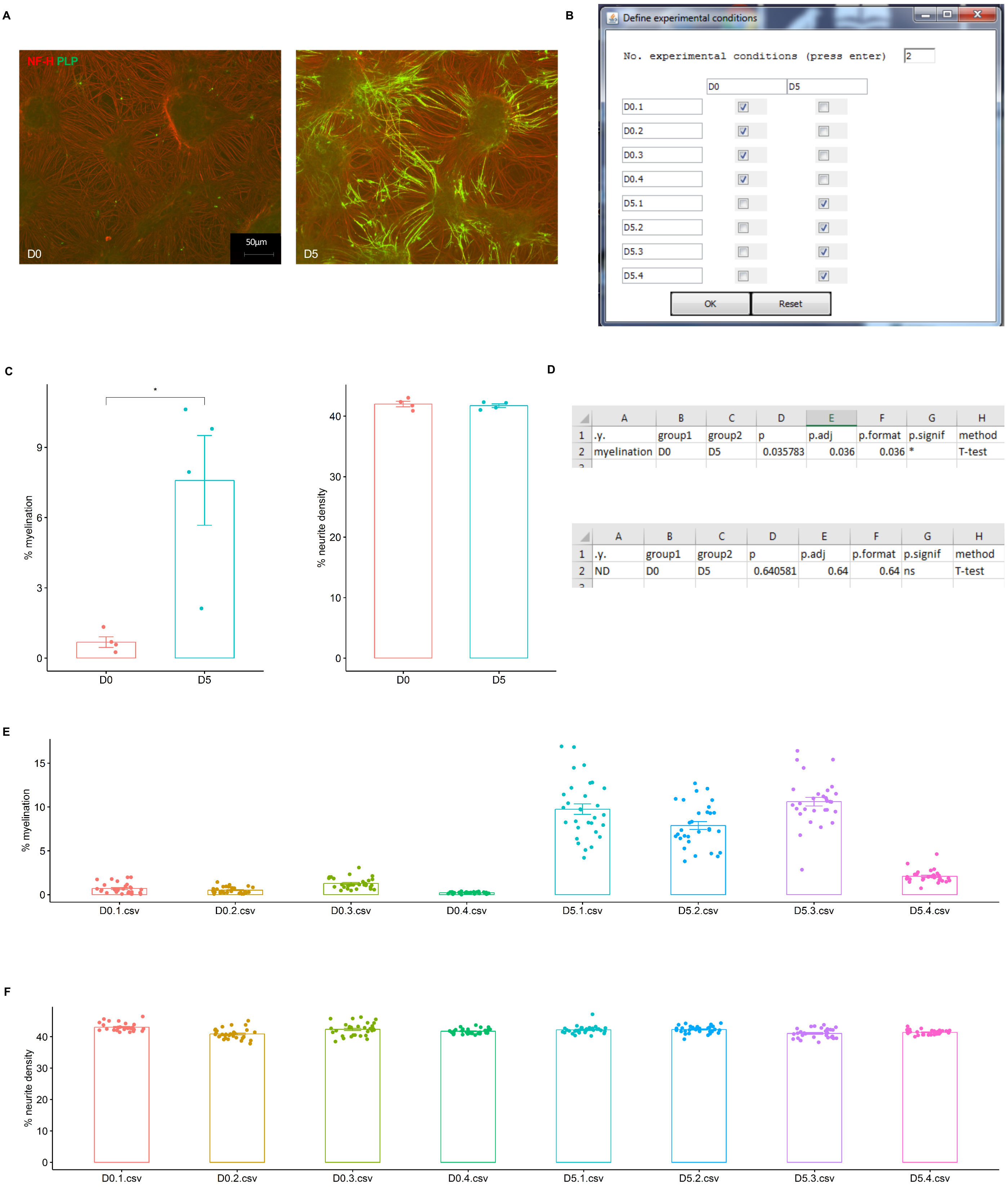

Supplementary figure 2: Automated statistical analysis using ggpubr. **A)** Rat myelinating cultures (DIV) after complement demyelination (DIV 0) and remyelination after 5 DIV (PLP green, NF-H red). **B)** MyelinJ dialog for entering comparisons. In this case 2 experimental conditions have been entered. The user then writes the name of each experimental condition in the text fields above each column. The checkboxes are used to define which experimental condition each experiment is in. **C)** The ggpubr package, in R, was used for graphs and statistical analysis. For each experiment the average % neurite density and % myelination is calculated and then a T test is used to compare between experimental conditions. For more than two experimental conditions the false discovery rate (FDR) is used for correcting multiple comparisons. **D**) CSV file containing results from the statistical analysis. The first being for % myelination and the second for % neurite density. **E)** % myelination calculated using PLP immunoreactivity as a percentage of NF-H immunoreactivity **F)** % neurite density calculated using NF-H immunoreactivity as a percentage of total pixels. Biological replicates = 4, technical = 3.
